# Predicting off-track development in infants aged 0–6 months in low-resource settings using machine learning

**DOI:** 10.1038/s41390-026-04761-7

**Published:** 2026-01-30

**Authors:** Faith Neema Benson, Rachel Odhiambo, Anthony K. Ngugi, Willie Brink, Akbar K. Waljee, Cheryl A. Moyer, Ji Zhu, Felix Agoi, Amina Abubakar

**Affiliations:** 1 Institute for Human Development, Aga Khan University, Nairobi, Kenya; 2 Department of Mathematical Sciences, Stellenbosch University, Stellenbosch, South Africa; 3 Department of Population Health, Medical College, Aga Khan University, Nairobi, Kenya; 4 Department of Learning Health Sciences, University of Michigan, Ann Arbor, MI USA; 5 Center for Global Health Equity, University of Michigan, Ann Arbor, MI USA; 6 Department of Statistics, University of Michigan, Ann Arbor, MI USA; 7 Environmental Health and Governance Centre, Africa International University, Nairobi, Kenya; 8 Department of Psychiatry, University of Oxford, Oxford, UK; 9 Neuroscience Unit, KEMRI-Wellcome Trust Research Programme, Kilifi, Kenya

## Abstract

**Background:**

This study aimed to address the critical gap in the limited application of machine learning (ML) for identifying developmental delays in low-resource settings by developing models to predict off-track development in infants aged 0 to 6 months and identify key predictors.

**Methods:**

A cross-sectional study involving 1,995 singleton infants aged 0 to 6 months was conducted in Kaloleni and Rabai sub-counties, Kilifi, Kenya, between March 2023 and March 2024. Development was assessed using the World Health Organization’s Indicators of Infant and Young Child Development tool, with Development-for-Age Z-scores used to classify infants as on- or off-track. Ridge logistic regression (LR), random forest (RF), and extreme gradient boosting (XGBoost) models were trained using sociodemographic, psychosocial, clinical/biological, nutritional, and health-related predictors. Performance was evaluated using area under the receiver operating characteristic curve (AUC), accuracy, sensitivity, and specificity. SHapley Additive exPlanations enhanced model interpretability.

**Results:**

Approximately 10.4% of infants were developmentally off-track. Ridge LR, RF, and XGBoost showed similar performance, with AUCs of 76.6%, 75.8%, and 76.1%, respectively. Limited psychosocial stimulation and increasing infant age were the strongest predictors.

**Conclusions:**

This study highlights the burden of developmental delays in low-resource settings. ML models

show promise for early risk prediction and targeted intervention, though further validation is

recommended.

**Impact:**

- Early intervention programs are proven to enhance optimal childhood development, yet the vital step of early identification of developmental delays is often overlooked.
- While machine learning is increasingly used to predict or identify health outcomes, its application in identifying developmental outcomes, particularly in low-resource settings, remains limited.
- This study contributes to the literature by applying machine learning to identify infants who are developmentally off-track and highlights key predictors. Limited psychosocial stimulation and increasing infant age were the strongest predictors, alongside low socioeconomic status, maternal mental health challenges, limited healthcare access, and nutritional and biological risks.

## Introduction

The earliest years of life, particularly from birth to age 3, represent a critical window for brain development, characterized by plasticity and sensitivity to environmental influences, both positive and negative.^1^ If foundational skills are not adequately nurtured during this sensitive period, children may face a permanent loss of developmental potential. This can lead to long-term negative consequences across the life course, including lower educational attainment, reduced social mobility, limited economic achievement, and poorer health outcomes.^1–3^ One of the aims of the Sustainable Development Goals is to increase the proportion of children under 5 years of age who are developmentally on track in terms of health, learning, and psychosocial well-being.^4^ Being developmentally on track means that children reach the milestones expected for their age across key functional domains, including motor, cognitive, language, and socio-emotional development. Despite this global priority, it is estimated that more than 250 million children in low- and middle-income countries are at risk of not reaching their full developmental potential.^5^ Sub-Saharan Africa (SSA) accounts for a significant share of this burden, contributing 29.6% of all children globally identified as living with developmental disabilities.^6^

While evidence supports the effectiveness of early intervention programs in promoting optimal childhood development,^2^ early identification remains a critical first step that has often been overlooked. Adverse developmental outcomes in childhood are complex, shaped by interactions among various factors such as nutrition, economic status, and the environment, yet the underlying mechanisms behind these outcomes remain poorly understood. Traditional statistical methods often rely on strong assumptions, such as linearity, which may not hold true in real-world data characterized by complexity, variability, and nonlinear relationships.^7^ In contrast, artificial intelligence methods, specifically machine learning (ML), can handle numerous variables and capture complex, nonlinear, and interactive patterns,^8^ enabling them to make accurate predictions in identifying outcomes. Despite the growing use of ML in predicting health outcomes,^9^ its application to predicting developmental outcomes at the population level remains largely unexplored in low-resource settings, particularly in SSA. Recognizing that interventions are more effective when implemented at the earliest stages of life, this study focuses on identifying infants who are developmentally off-track in the first six months of life in a low-resource setting. It does so by leveraging a combination of social demographic, economical, nutritional, clinical/biological, psychosocial, mental and health-related factors. The study aimed to develop ML models to predict off-track development in infants aged 0 to 6 months and identify key predictors influencing this outcome. These insights can inform early interventions that support optimal child development and help children reach their full developmental potential in low-resource settings.

## Methods

This study was conducted to predict off-track development in infants aged 0–6 months and identify key predictors influencing this outcome. These insights are intended to inform early interventions aimed at improving developmental outcomes in low-resource settings. The following sections outline the specific procedures and techniques used throughout the study.

### Study setting and participants

This research took place in Kaloleni and Rabai, two rural sub-counties located in Kilifi County along Kenya’s coast. These areas are among the most economically disadvantaged in the country.^10^ The region has an estimated population of 350,000 individuals living in about 47,000 households. Access to healthcare is limited, with only 40 health facilities serving a widely dispersed population. The region faces significant poverty, with around 70% of residents living below the national poverty line, and the majority, about 81%, depending on small-scale farming, craftwork, informal labor, and petty trade for income. Moreover, maternal and child health outcomes in this region are significantly poorer than national averages.^11^

This study involved 1,995 singleton infants between the ages of 0 and 6 months who were healthy at the time of data collection, with no reported symptoms such as fever or diarrhea. Participants were identified by Community Health Extension Workers (CHEWs) using the Kaloleni-Rabai Health and Demographic Surveillance System (KRHDSS). The system is managed by the Department of Population Health (DPH) at the Aga Khan University, Nairobi, Kenya.^12^ It is a population-based registry that monitors ~21,000 households in the area and has conducted annual data collection since 2017.^12^

### Data collection and study tools

From March 2023 to March 2024, a cross-sectional study was conducted involving 2085 children. This included 1995 singleton infants and 35 pairs of twins with their biological mothers, as well as 20 singleton infants under the care of non-biological guardians, typically due to the absence or passing of the biological mother. Eligible participants were identified at the household level with the assistance of CHEWs and Community Health Promoters (CHPs).

Data collection teams, consisting of trained enumerators from the Institute for Human Development at the Aga Khan University, in collaboration with CHPs, visited participants’ homes to collect data. Written informed consent was obtained from caregivers or mothers for their own and their child’s participation. Data was gathered using tablets equipped with Open Data Kit software, through interviewer-administered questionnaires as described below.

The study utilized several validated tools to collect data. These included: the World Health Organization’s Indicators of Infant and Young Child Development (WHO-IYCD), a caregiver-report questionnaire assessing motor, language, cognitive, socio-emotional, and behavioral functioning;^13^ the Patient Health Questionnaire, for maternal depressive symptoms^14^; and the Generalized Anxiety Disorder Scale, for maternal anxiety symptoms.^15^ The Home Screening Questionnaire was used to assess psychosocial stimulation in the home environment.^16^ Socioeconomic status was measured using a tool adapted from,^17^ and maternal and child health history was obtained through a yes/no parent-report questionnaire. Finally, anthropometric measurements (height and weight) for both mothers and children were collected following WHO-recommended procedures. Detailed information about these tools is provided in the Supplementary Text S1.

### Data analysis

#### Outcome variable

The outcome variable in this study was overall child development status, categorized as either “developmentally off-track” or “typical developing.” Development status was assessed using Development-for-Age Z-scores (DAZ), calculated from WHO-IYCD scores and the child’s age in years. DAZ values were generated using an Excel tool provided in the WHO IYCD Instrument Manual (version 1.2, Web Appendix VII),^13^ which automatically computed Z-scores based on the child’s age and raw IYCD scores. In line with WHO 2005 reference criteria, scores < −2 were classified as “developmentally off-track,” while scores ≥ −2 were considered “typical developing.” The internal consistency of the WHO-IYCD scale was acceptable, with a Cronbach’s alpha of 0.91 (95% CI: 0.905–0.916).

#### Data preparation and exploratory data analysis

The data were analyzed using Python (version 3.11.3).^18^ The dataset included 64 predictive features selected based on prior research and input from domain expert (Supplementary Table S1). Given the minimal missing data (Supplementary Table S1), imputation was performed using logical reasoning and statistical methods, including median and mode, to ensure consistency and accuracy. For numerical variables, missing values were imputed using the median to maintain consistency and reduce the influence of potential outliers. For categorical variables, missing entries were imputed using the most frequent category. Descriptive statistics were used to summarize maternal and child characteristics, including proportions for categorical variables and medians with interquartile ranges for continuous variables. Group comparisons were performed using Pearson’s *χ*² test or Fisher’s exact test for categorical variables, and the Kruskal-Wallis test for non-normally distributed continuous data. The TableOne package in Python was used, with the *‘htest_name = True’* option to display the names of the statistical tests applied. Fisher’s Exact Test was automatically applied for categorical variables when appropriate, and Pearson’s *χ*² test otherwise. All hypothesis testing was two-sided, with statistical significance defined as a *p* < 0.05. Pearson correlation coefficients were calculated and visualized pairwise for all continuous features considered important or tentative, to inform feature selection based on expert judgment and model performance impact. For categorical variables, pairwise Cramér’s V correlations were assessed, with selection also guided by domain expertise. One-hot encoding was applied to categorical variables to facilitate model interpretability (Supplementary Table S2).

#### Model development and feature selection

This study evaluated three supervised ML models (classifiers): the ridge Logistic Regression (LR) and the advanced Random Forest (RF) and Extreme Gradient Boosting (XGBoost), chosen for their robust performance, widespread application in health-related studies, and ease of interpretation.^19,20^ The ridge LR model served as a baseline to assess consistency in findings and to evaluate whether the advanced models (RF and XGBoost) could enhance predictive performance by capturing nonlinear relationships among features. The models were implemented using the *sklearn.linear_model* library for LR, *sklearn.ensemble* for RF, and the *xgboost* library for XGBoost. To maintain class balance in both training and testing subsets, the dataset was stratified by the outcome variable and split using an 80:20 ratio, 80% for training and 20% for testing. A formal sample size calculation was not performed due to the exploratory nature of this predictive modeling study. However, we followed recommended ML practices, ensuring sufficient outcome events per predictor variable (EPV), with the training set exceeding the commonly accepted 10–20 EPV threshold to reduce overfitting.^21^ Feature scaling using *StandardScaler* was applied only to the LR model to ensure proper convergence and coefficient interpretation. Scaling was not applied to the RF and XGBoost models, as these tree-based algorithms are not sensitive to feature scaling. To address class imbalance, the RF and LR models incorporated the *class_weight* = *‘balanced’* parameter, which assigns weights inversely related to class frequencies, ensuring more equitable model predictions.^22^ For XGBoost, the *scale_pos_weight* parameter was set based on the ratio of positive to negative class samples, helping the model focus appropriately on the minority class.^23^ Hyperparameter tuning was conducted using a 10-fold cross-validation approach on the training data. In each iteration, 90% of the data was used for training and 10% for validation.^24^ A grid search strategy was applied to identify the best model configurations. Bootstrapping with 1000 resamples (*n* = 1000) was used to estimate the stability and variability of model performance metrics.^25^ Model performance was assessed using the area under the receiver operating characteristic curve (AUC), a key metric for evaluating classifier performance in the context of class imbalance.^26^

Feature selection was informed by importance scores derived from SHapley Additive exPlanations (SHAP), based on the method proposed by Lundberg SM, Erion GG and Lee S-I.^27^ Unlike conventional approaches such as gain-based importance used in tree-based models, SHAP offers a more robust and consistent estimation of each feature’s influence. Grounded in game theory principles, SHAP attributes a value to each feature that quantifies its unique contribution to the model’s prediction, providing a fair and interpretable representation of feature impact.

#### Model performance evaluation and interpretation

The final evaluation served as internal validation, as the test data originated from the same dataset. Once the best-performing model was identified, its effectiveness was tested using the reserved 20% of the data, which had not been involved in training or validation. This testing phase assessed the model’s ability to generalize to new, unseen data. A range of performance metrics, such as accuracy, sensitivity, and specificity, was used to provide a well-rounded evaluation of the model.^28^ To determine the optimal threshold for classification, the Youden Index was applied.^29^ Additionally, the AUC was computed to evaluate the model’s discriminatory power across different thresholds.^30^ Bootstrapping with 1000 resamples (*n* = 1000) was used to estimate the stability and variability of model performance metrics.

To improve the interpretability of the models during internal validation, SHAP values were employed to assess both feature importance and the contribution of individual features to model predictions. Two types of SHAP visualizations were utilized: dependence plots, which show the effect of specific features on model outcomes, and summary plots (such as bar and beeswarm plots), which provide an overview of feature importance. These visualizations were generated using the test dataset to assess the role and impact of each feature.

## Results

Table 1 presents the maternal and child characteristics included in the study. The median age of the children was 2 months (Q1: 1.0, Q3: 4.0), while the median age of the mothers was 27 years (Q1: 23.0, Q3: 32.0). A total of 1995 children were included in the study. Of these, 207 (10.4%) were identified as developmentally off-track, while 1788 (89.6%) were on-track.

**Table 1:** Maternal and child characteristics included in the study.

Variable	Overall *N* = 1995	Developmentally off-track		*P* value
		No *n* (%)	Yes *n* (%)	
		1788 (89.6%)	207 (10.4%)	
Child BMI z-scores, median [Q1, Q3]	0.1 [−0.7, 0.9]	0.1 [−0.7, 0.9]	−0.2 [−0.9, 0.7]	0.014^a^
Child head circumference z-scores, median [Q1, Q3]	0.2 [−0.5, 1.1]	0.2 [−0.5, 1.1]	0.3 [−0.6, 1.3]	0.699^a^
Number of children aged 1–5 years, median [Q1, Q3]	1.0 [0.0, 1.0]	1.0 [0.0, 1.0]	1.0 [0.0, 1.0]	0.063^a^
Total number of children, median [Q1, Q3]	3.0 [1.0, 4.0]	3.0 [1.0, 4.0]	3.0 [1.0, 5.0]	0.043^a^
Number of pregnancies, median [Q1, Q3]	3.0 [2.0, 5.0]	3.0 [2.0, 5.0]	3.0 [2.0, 5.0]	0.100^a^
Maternal age (years), median [Q1, Q3]	27.0 [23.0, 32.0]	27.0 [23.0, 32.0]	27.0 [23.0, 33.0]	0.851^a^
Child birthweight (kg), median [Q1, Q3]	3.0 [2.7, 3.3]	3.0 [2.7, 3.3]	3.0 [2.6, 3.2]	0.020^a^
Maternal BMI, median [Q1, Q3]	21.2 [19.3, 24.0]	21.3 [19.3, 24.0]	20.8 [19.3, 23.5]	0.119^a^
Maternal depressive symptom scores, median [Q1, Q3]	0.0 [0.0, 3.0]	0.0 [0.0, 3.0]	1.0 [0.0, 3.0]	0.168^a^
Maternal anxiety symptom scores, median [Q1, Q3]	0.0 [0.0, 1.0]	0.0 [0.0, 1.0]	0.0 [0.0, 1.0]	0.840^a^
Child age (months), median [Q1, Q3]	2.0 [1.0, 4.0]	2.0 [1.0, 4.0]	3.0 [1.0, 5.0]	<0.001^a^
Child weight-for-age z-scores, median [Q1, Q3]	−0.3 [−1.1, 0.4]	−0.3 [−1.1, 0.4]	−0.5 [−1.3, 0.2]	0.011^a^
Child height-for-age z-scores, median [Q1, Q3]	−0.7 [−1.6, 0.1]	−0.7 [−1.6, 0.1]	−0.8 [−1.6, 0.1]	0.533^a^
Child weight-for-height z-scores, median [Q1, Q3]	0.4 [−0.2, 1.3]	0.4 [−0.2, 1.3]	0.4 [−0.5, 1.1]	0.012^a^
Wealth index, median [Q1, Q3]	−0.2 [−0.3, 0.1]	−0.2 [−0.3, 0.2]	−0.2 [−0.3, 0.0]	0.222^a^
Home environment quality scores, median [Q1, Q3]	13.0 [12.0, 15.0]	14.0 [12.0, 15.0]	13.0 [11.0, 14.0]	<0.001^a^
Married	1770 (88.7)	1580 (88.4)	190 (91.8)	
Unmarried	225 (11.3)	208 (11.6)	17 (8.2)	
Maternal education, *n* (%)				
No education	317 (15.9)	271 (15.2)	46 (22.2)	0.002^b^
Primary	1262 (63.3)	1128 (63.1)	134 (64.7)	
Secondary and above	416 (20.9)	389 (21.8)	27 (13.0)	
Delivery assistant, *n* (%)				0.002^b^
Doctor	608 (30.5)	567 (31.7)	41 (19.8)	
Nurse	1165 (58.4)	1018 (56.9)	147 (71.0)	
Relative	143 (7.2)	129 (7.2)	14 (6.8)	
TBA	42 (2.1)	39 (2.2)	3 (1.4)	
Unassisted	37 (1.9)	35 (2.0)	2 (1.0)	
Mother enrolled in any program or project, *n* (%)				
No	1978 (99.1)	1771 (99.0)	207 (100.0)	0.245^c^
Yes	17 (0.9)	17 (1.0)		
Mother caring for children with disabilities, *n* (%)				
No	1964 (98.4)	1762 (98.5)	202 (97.6)	0.246^c^
Yes	31 (1.6)	26 (1.5)	5 (2.4)	
Mother lives with partner, *n* (%)				0.217^b^
No	283 (14.2)	260 (14.5)	23 (11.1)	
Yes	1712 (85.8)	1528 (85.5)	184 (88.9)	
Partner’s education, *n* (%)				0.579^b^
No education	113 (5.7)	98 (5.5)	15 (7.2)	
Primary	1347 (67.5)	1209 (67.6)	138 (66.7)	
Secondary and above	535 (26.8)	481 (26.9)	54 (26.1)	
Mother had pregnancy complications, *n* (%)				0.658^b^
No	1777 (89.1)	1595 (89.2)	182 (87.9)	
Yes	218 (10.9)	193 (10.8)	25 (12.1)	
Mother attended antenatal clinic, *n* (%)				0.664^b^
No	94 (4.7)	86 (4.8)	8 (3.9)	
Yes	1901 (95.3)	1702 (95.2)	199 (96.1)	
Place of delivery, *n* (%)				<0.001^b^
Clinic	103 (5.2)	68 (3.8)	35 (16.9)	
Home	218 (10.9)	199 (11.1)	19 (9.2)	
Hospital	1674 (83.9)	1521 (85.1)	153 (73.9)	
Mother had delivery problems, *n* (%)				0.943^b^
No	1882 (94.3)	1686 (94.3)	196 (94.7)	
Yes	113 (5.7)	102 (5.7)	11 (5.3)	
Child cried after delivery, *n* (%)				0.362^b^
No	95 (4.8)	82 (4.6)	13 (6.3)	
Yes	1900 (95.2)	1706 (95.4)	194 (93.7)	
Child had problems after delivery, *n* (%)				0.181^b^
No	1910 (95.7)	1716 (96.0)	194 (93.7)	
Yes	85 (4.3)	72 (4.0)	13 (6.3)	
Child had problems in first 30 days, *n* (%)				0.101^b^
No	1861 (93.3)	1674 (93.6)	187 (90.3)	
Yes	134 (6.7)	114 (6.4)	20 (9.7)	
Mother had healthcard, *n* (%)				0.796^c^
No	42 (2.1)	37 (2.1)	202 (97.6)	
Yes	1953 (97.9)	1751 (97.9)	18 (8.7)	
Child received immunization, *n* (%)				1.000^b^
No	175 (8.8)	157 (8.8)	18 (8.7)	
Yes	1820 (91.2)	1631 (91.2)	189 (91.3)	
Child admitted to hospital previously, *n* (%)				0.278^b^
No	1945 (97.5)	1746 (97.7)	199 (96.1)	
Yes	50 (2.5)	42 (2.3)	8 (3.9)	
Child gender, *n* (%)				0.143^b^
Female	1046 (52.4)	927 (51.8)	119 (57.5)	
Male	949 (47.6)	861 (48.2)	88 (42.5)	
Maternal religion, *n* (%)				0.005^b^
Christian	1435 (71.9)	1280 (71.6)	155 (74.9)	
Islam	461 (23.1)	426 (23.8)	35 (16.9)	
Other	8 (0.4)	5 (0.3)	3 (1.4)	
Traditional	91 (4.6)	77 (4.3)	14 (6.8)	

^a^Refers to the Kruskal-Wallis test.

^b^Refers to Pearson’s *χ*².

^c^Refers to Fisher’s exact test.

Significant differences between groups were found for some characteristics (*p* < 0.05; Table 1). Correlation matrices for both numerical and categorical variables are presented in Supplementary Figs. S1 and S2. Among the numerical variables, a strong correlation was observed between the number of children and the number of pregnancies (*r* = 0.96). Both variables were retained in the analysis following expert recommendations, as they capture different dimensions of reproductive history: the number of children refers to all currently living children, whereas the number of pregnancies accounts for all pregnancies, including both live births and miscarriages. Although child BMI z-scores and weight-for-height z-scores were highly correlated (*r* = 0.84), both were kept due to their unique contributions to the analysis. Similarly, despite a strong correlation between living with a partner and marital status (*r* = 0.87) both features were included based on expert guidance.

### Model performance evaluation

Table 2 summarizes the performance of all three models, each achieving AUCs above 0.75 on unseen test data using 43 features. All three models performed similarly in predicting off-track development, with ridge LR having an AUC of 0.766 (95% CI: 0.682–0.841), RF an AUC of 0.758 (95% CI: 0.672–0.833), and XGBoost an AUC of 0.761 (95% CI: 0.675–0.835). Additional performance metrics, including accuracy, sensitivity, and specificity, are also presented in the table. When using a reduced set of the 20 most important features selected via the SHAP method, model performance remained stable. LR achieved an AUC of 0.773 (95% CI: 0.689–0.847), with XGBoost at 0.760 (95% CI: 0.678–0.833) and RF at 0.758 (95% CI: 0.677–0.833).

**Table 2:** Evaluation of model performance on unseen test data

Outcome variable	Algorithm	No of features	Test size	Best cutoff	Sensitivity (95%CI)	Specificity (95%CI)	AUC (95%CI)	Accuracy (95%CI)
Off-track development	Ridge logistic regression	43	399 (20%)	0.497	0.732 [0.727–0.736]	0.670 [0.669–0.672]	0.766 [0.682, 0.841]	0.677 [0.675–0.678]
		20	399 (20%)	0.492	0.732 [0.727– 0.736]	0.665 [0.663–0.666]	0.773 [0.689, 0.847]	0.672 [0.670- 0.673]
	XGBoost	43	399 (20%)	0.500	0.683 [0.678–0.688]	0.696 [0.694–0.697]	0.761 [0.675, 0.835]	0.694 [0.693–0.696]
		20	399 (20%)	0.500	0.683 [0.678–0.688]	0.696 [0.694–0.697]	0.760 [0.678, 0.833]	0.694[0.693–0.696]
	Random forest	43	399 (20%)	0.468	0.634 [0.629–0.639]	0.757 [0.756–0.758]	0.758 [0.672, 0.833]	0.744 [0.743- 0.746]
		20	399 (20%)	0.475	0.634 [0.629–0.639]	0.749 [0.747–0.750]	0.758 [0.677, 0.833]	0.737 [0.736–0.738]

### Model interpretation

**Fig. 1:**
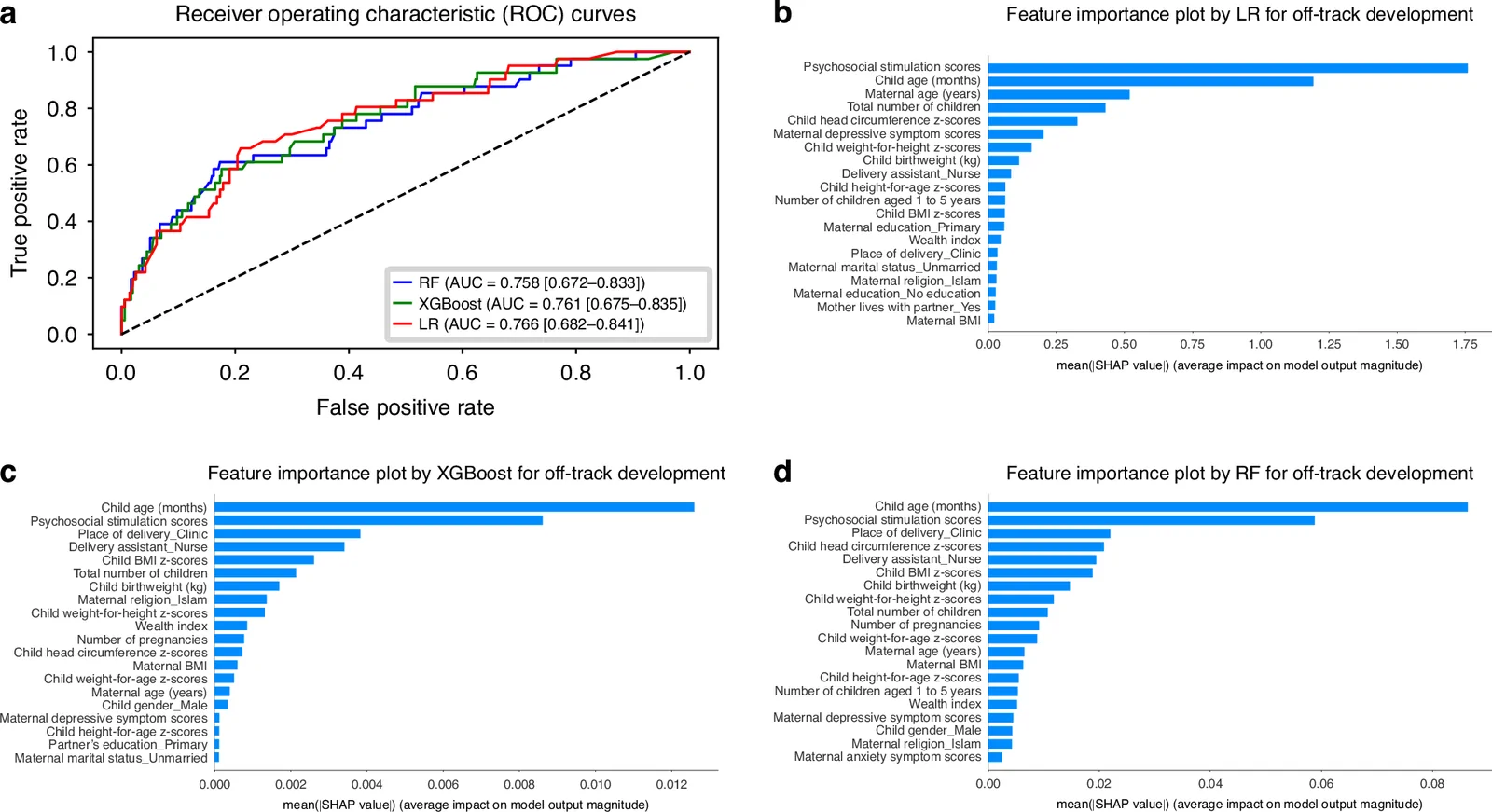
Model performance and important predictors of off-track development. **a** presents the Receiver Operating Characteristic (ROC) curves for three models, ridge Logistic Regression (LR), Random Forest (RF) and Extreme Gradient Boosting (XGBoost) along with their corresponding AUC values for predicting off-track development in infants aged 0–6 months. SHAP feature importance **b**–**d** display the top 20 important predictors of off-track development ranked from most to least important.

Figure 1 presents the Receiver Operating Characteristic (ROC) curves and feature importance plots generated using all 43 features.

**Fig. 2:**
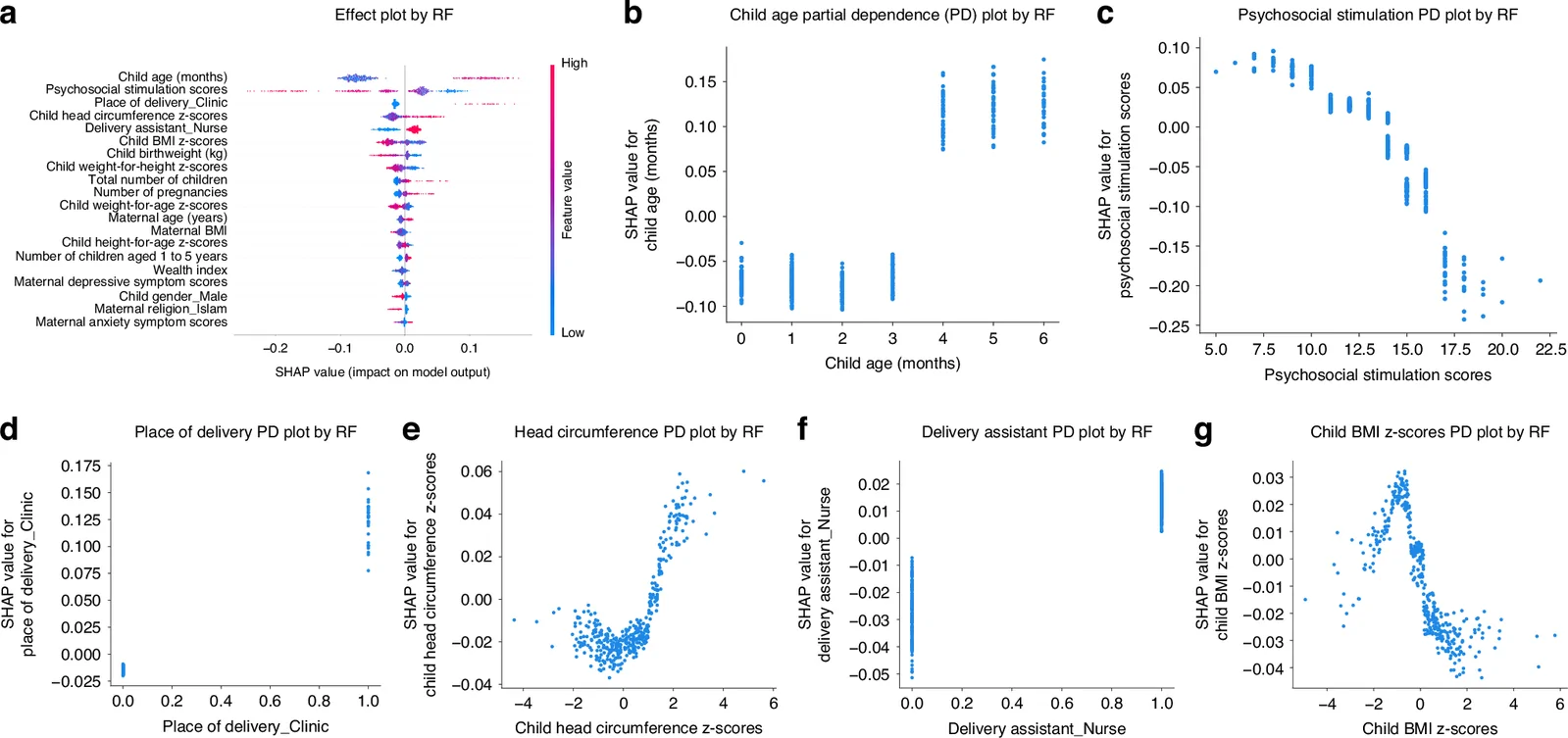
Key predictors of off-track development identified by Random Forest. **a** An effect plot, (SHAP beeswarm plot) shows the top 20 important predictors of off-track development from a Random Forest (RF) model, ranked by importance. Color indicates the feature value (red = high, blue = low), and position along the x-axis shows the direction and strength of impact. Red (high) values of a predictor on the right indicate that higher values increase the risk of off-track development, while blue (low) values on the right suggest that lower values are also associated with higher risk. The opposite applies for predictors on the left side of the plot. **b**–**g** Display the SHAP partial dependence for the top 6 key predictors. Increased child age limited psychosocial stimulation, delivery at a clinic, larger head circumference, assistance by a nurse during delivery, and low child BMI are all positively associated with being developmentally off-track.

**Fig. 3:**
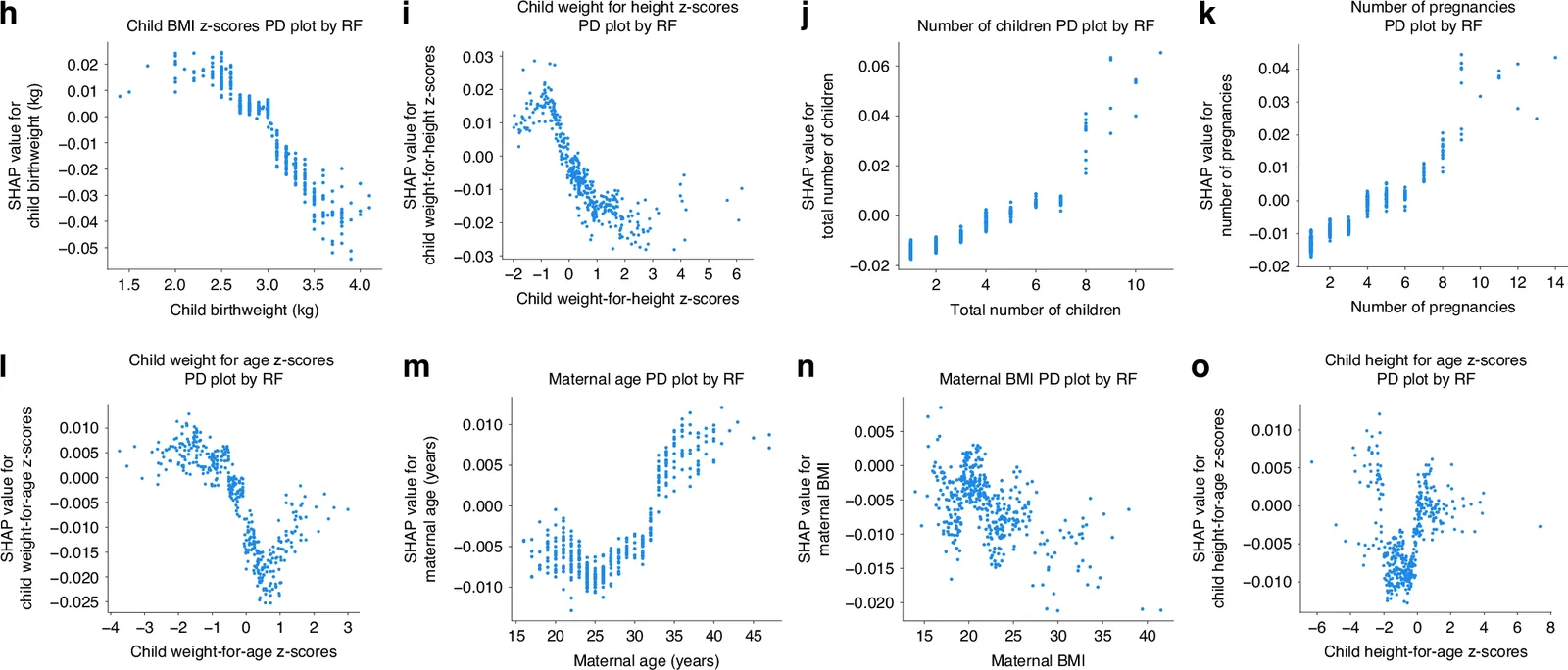
Additional key predictors of off-track development identified by Random Forest. **h**–**o** Display the SHAP partial dependence for the 7th to 14th most important predictors. Low child birthweight, low weight-for-height, a higher number of children per mother, more maternal pregnancies, low weight-for-age, older maternal age, low maternal BMI, and short height-for-age are all positively associated with off-track development.

Higher infant age and limited psychosocial stimulation scores consistently emerged as a critical predictor across all models. Other key predictors among the top 20 important features included socioeconomic and demographic factors (low household wealth; increased maternal age, number of children, number of children aged 1–5 years, number of pregnancies, maternal education [no education], partner’s education [primary], religion [Islam], marital status [unmarried], and not living with a partner); nutritional indicators (child’s low weight-for-height, weight-for-age, height-for-age, BMI z-scores and maternal BMI); maternal mental health problems (depression and anxiety); health system–related factors (place of delivery [private clinics] and delivery assistant [nurses]); and clinical/biological factors (larger head circumference and low birthweight). Figures 2, 3*and* Supplementary Fig. S3 illustrate the magnitude, direction of effects, and impact of these predictors on off-track development. RF plots was selected for its interpretability and its ability to capture nonlinear relationships, which can be visualized using partial dependence plots. XGBoost produced similar plots.

## Discussion

This study used ML techniques to predict off-track development in infants aged 0–6 months. The findings highlight the significant burden of not reaching full developmental potential in low-resource settings and underscore critical areas for targeted interventions. It also demonstrates the potential of leveraging advanced technology for effective modeling and prediction.

We found that 10.4% of children in our study population were developmentally off-track, which is more than twice the global prevalence estimate of 4.3%,^6^ and higher than the SSA estimate of 6%.^6^ These findings highlight a substantial burden of poor developmental outcomes in our setting. The observed prevalence is comparable to that reported in a population-based study from Uganda, where 12.7% of infants had neurodevelopmental delays.^31^ Compared to our findings, another study conducted in an informal settlement in Kenya reported a lower prevalence of 8.7% among children aged 0–48 months.^32^ The relatively higher prevalence observed in our study may be attributed to differences in the age range of children assessed, the developmental domains evaluated, the low socioeconomic status of the population, parental challenges such as limited access to quality healthcare services, and the measurement tools used. These findings align with broader concerns about early childhood development in low-resource settings and emphasize the need for early identification, intervention, and investment in improved early childhood development services.

All models achieved comparable and acceptable performance,^33^ with AUC scores above 0.75 in distinguishing infants who were developmentally off-track from those who were typically developing. While previous studies from high-income countries have predicted developmental outcomes such as cognitive functioning at later ages (2–6 years) using larger samples exceeding 3000 participants,^34–36^ our study applied ML methods to a smaller cohort of 1995 children assessed between 0 and 6 months of age. Despite these differences, our findings are consistent with those studies, which reported AUCs of ~0.70, suggesting that meaningful prediction of developmental risk is feasible even during early infancy. These findings suggest that ridge LR performs well even with a moderate sample size and has practical potential for integration into early childhood development services to facilitate screening programs. However, with larger datasets, more complex models such as RF and XGBoost may offer added advantages by capturing nonlinear relationships and interactions among predictors. In addition to AUC, the models performed comparably across accuracy, sensitivity, and specificity metrics. Importantly, predictive performance remained robust when using only the top 20 SHAP-ranked features, indicating that reliable predictions can be made with a more concise feature set. To the best of our knowledge, no prior studies globally have applied predictive ML models to identify children who are developmentally off-track at such an early stage, making this the first attempt to explore this potential in our context.

In addition to predictive modeling, we identified the top 20 most important predictors (key predictors) from each model. The findings highlight the complex interplay of factors linked to developmental delays. These include socioeconomic and demographic variables such as low household wealth; increased maternal age, number of children, number of pregnancies, child’s age, and the number of children aged 1–5 years; low levels of maternal and partner education; unmarried status; Islamic religion; and not living with a partner. Nutritional risks were also evident, including low child weight-for-height, weight-for-age, height-for-age, BMI z-scores, and low maternal BMI. Additional contributors were maternal mental health problems (depression and anxiety), health system–related factors (delivery in clinics and assistance by nurses), clinical/biological indicators (larger head circumference and low birthweight), and limited psychosocial stimulation. Although not all 20 key predictors are routinely collected in standard practice, several, such as child age and birthweight, are commonly recorded, while others, including psychosocial support and maternal mental health status, would need to be collected specifically for prediction purposes.

These findings are consistent with existing literature, which indicates that multiple early risk factors contribute to greater vulnerability for adverse developmental outcomes. Studies show that the more risk factors a child is exposed to, the poorer their developmental outcomes tend to be.^37^ Notably, limited psychosocial stimulation consistently emerged as a strong predictor across all models, highlighting the importance of prioritizing interventions that promote early psychosocial stimulation for children in comparable settings. Furthermore, our findings show that even within the 0–6 months window, older infants were more likely to be off-track. However, this should be interpreted with caution, as age was included as a predictor to account for expected developmental variability, as reported in previous studies.^38,39^

Interestingly, place of delivery (clinic) emerged as one of the critical predictors of off-track development, with these clinics being privately owned. This may be partly attributed to many mothers opting to deliver at the nearest private clinics due to pregnancy-related problems. Additionally, the limited availability of equipment and personnel in private clinics can hinder the effective management of childbirth complications. For instance, many private clinics may lack essential resources such as oxygen or surgical facilities, underscoring the importance of timely referrals to better-equipped centers in emergency situations. Additionally, deliveries assisted by nurses were also linked to increased risk; however, this finding should be interpreted cautiously, as it may be confounded by the place of delivery and does not necessarily indicate a direct causal effect, Supplementary Fig. S4. Other variables, such as religion, marital status, and living with a partner, may also function as confounding factors, interacting with the most important predictors in the model.

This study utilized advanced ML techniques to accurately predict off-track development in infants, leveraging ML’s ability to capture complex, nonlinear relationships between predictors, in contrast to traditional statistical models that combine risk factors additively, assuming independent effects. In addition, we incorporated a wide range of relevant predictors, including socioeconomic, demographic, clinical/biological, nutrition, mental health, psychosocial stimulation, and other health-related factors, ensuring a comprehensive and contextually relevant approach to early screening in low-resource settings. Our findings demonstrate that ML can effectively screen infants at an early age in low-resource settings, identifying with off-track development and informing targeted interventions to support children in reaching their full developmental potential. The application of SHAP for feature importance and effect analysis enhanced model interpretability, offering actionable insights for policymakers and practitioners, as illustrated in Figs. 2, 3*, and* Supplementary Fig. S3.

However, this study has some limitations. The moderate sample size and imbalanced dataset may limit the predictive power and generalizability of the ML models. A key ethical consideration is the identification of children as being developmentally off-track on data collected during a single early developmental phase (0–6 months), which may lead to stigma or unintended consequences associated with early labeling. Assessing children at such an early age may also limit the ability to predict long-term developmental outcomes, as early assessments often correlate less strongly with later performance, and we acknowledge this as a significant limitation of the current study. However, this study is part of an ongoing cohort, and we plan to follow up with these children in the long term to enhance understanding of developmental trajectories and validating the predictive models across diverse populations and advanced age groups. Following this validation, we aim to develop a diagnostic or screening app for use in frontline healthcare settings. Additionally, data collected through caregiver-reported questionnaires may be subject to reporting bias, particularly underreporting due to social stigma.

## Conclusion

This study shows that even in early infancy, there is a high burden of children who are developmentally off-track, indicating a need for early intervention to support optimal growth and help children reach their full developmental potential. Limited psychosocial stimulation and increasing infant age were the strongest predictors, emphasizing the importance of interventions that prioritize enriching caregiving environments to promote psychosocial stimulation. Complementary interventions, such as improving socioeconomic conditions, supporting maternal mental health, enhancing healthcare access, and addressing nutrition and biological risks, could also play a key role in supporting child development. ML techniques show promise for improving risk prediction and targeted support. As a next step, we plan to conduct a longitudinal study to monitor developmental trajectories over time and validate the predictive models across diverse populations and advanced age groups. Following validation, we aim to develop a diagnostic or screening app for use in frontline healthcare settings.

## Supplementary information

Supplementary material 26112025

## Data Availability

The data supporting the findings of this study are not publicly available due to confidentiality and ethical restrictions. However, they may be made available by the corresponding author upon reasonable request and with appropriate approvals.
