# Supplementary material for: Predicting off-track development in infants aged 0–6 months in low-resource settings using machine learning
Source: Pediatr Res. 2026 Jan 30;100(3):1100–8. doi: 10.1038/s41390-026-04761-7 (PMC13672098; doi:10.1038/s41390-026-04761-7)
Supplement: Supplementary file 1 [file 41390_2026_4761_MOESM1_ESM.pdf]

Table of contents

1. **Supplementary Table S1: Potential features considered for the modelling process and level of missingness** 2
2. **Supplementary Figure S1: Correlation of numerical features** 4
3. **Supplementary Figure S2: Correlation of categorical features** 5
4. **Supplementary Figure S3: SHAP partial dependence plots for the 15th to 20th most important predictors, by Random Forest (RF)** 6
5. **Supplementary Figure S4: Interaction between place of delivery (clinic) and delivery assistant (nurse)** 7
6. **Supplementary Table S2: Features retained for the modelling process after one hot encoding** 7
7. **Supplementary Text S1: Study tools** 8
   - **7.1. World Health Organisation Indicators of Infant and Young Child Development (WHO-IYCD)** 8
   - **7.2. Patient Health Questionnaire (PHQ-9)** 8
   - **7.3. Generalised Anxiety Disorder (GAD-7)** 8
   - **7.4. Home Screening Questionnaire (HSQ)** 8
   - **7.5. Social demographic and economic questionnaire** 8
   - **7.6. Clinical and health history questionnaire** 9
   - **7.7. Anthropometric measurements** 9
8. **Reference** 9

1. Supplementary Table S1: Potential features considered for the modelling process and level of missingness

Feature	Categories	Feature type	N (%) missing
Child BMI z-scores		Numerical	0 (0)
Child head circumference z-scores		Numerical	0 (0)
Number of children aged 1 to 5 years		Numerical	1 (0·05)
Total number of children		Numerical	1 (0·05)
Number of pregnancies		Numerical	0 (0)
Maternal age (years)		Numerical	0 (0)
Child birthweight (kg)		Numerical	0 (0)
Maternal BMI		Numerical	0 (0)
Maternal depressive symptom scores		Numerical	0 (0)
Maternal anxiety symptom scores		Numerical	0 (0)
Child age (months)		Numerical	0 (0)
Child weight-for-age z-scores		Numerical	0 (0)
Child height-for-age z-scores		Numerical	0 (0)
Child weight-for-height z-scores		Numerical	0 (0)
Wealth index		Numerical	0 (0)
Psychosocial stimulation scores		Numerical	0 (0)
Marital status	Married<br>Unmarried	Categorical (derived)	0 (0)
Maternal education	No education<br>Primary<br>Secondary and above	Categorical (derived)	0(0)
Delivery assistant	Doctor<br>Nurse<br>Relative<br>TBA<br>Unassisted	Categorical	8 (0·40)
Mother enrolled in any program or project	No<br>Yes	Categorical	7 (0·35)
Mother caring for children with disabilities	No<br>Yes	Categorical	
Mother lives with partner	No<br>Yes	Categorical	225 (11·28)
Partner's education	No education<br>Primary<br>Secondary and above	Categorical (derived)	0(0)
Mother had pregnancy complications	No<br>Yes	Categorical	8 (0·40)
Mother attended antenatal clinic	No<br>Yes	Categorical	8 (0·40)
Place of delivery	Clinic<br>Home<br>Hospital	Categorical	8 (0·40)

Mother had delivery problems	No<br>Yes	Categorical	8 (0·40)
Child cried after delivery	No<br>Yes	Categorical	0(0)
Child had problems after delivery	No<br>Yes	Categorical	0(0)
Child had problems in first 30 days	No<br>Yes	Categorical	0(0)
Mother had health card	No<br>Yes	Categorical	0(0)
Child received immunization	No<br>Yes	Categorical	0(0)
Child admitted to hospital previously	No<br>Yes	Categorical	0(0)
Child gender	Female<br>Male	Categorical	0(0)
Maternal religion	Christian<br>Islam<br>Traditional<br>Other (e.g. Atheists)	Categorical	0(0)

Legend: BMI, Body Mass Index; Kg, Kilograms; TBA, Traditional Birth Attendant

2. **Supplementary Figure S1: Correlation of numerical features**

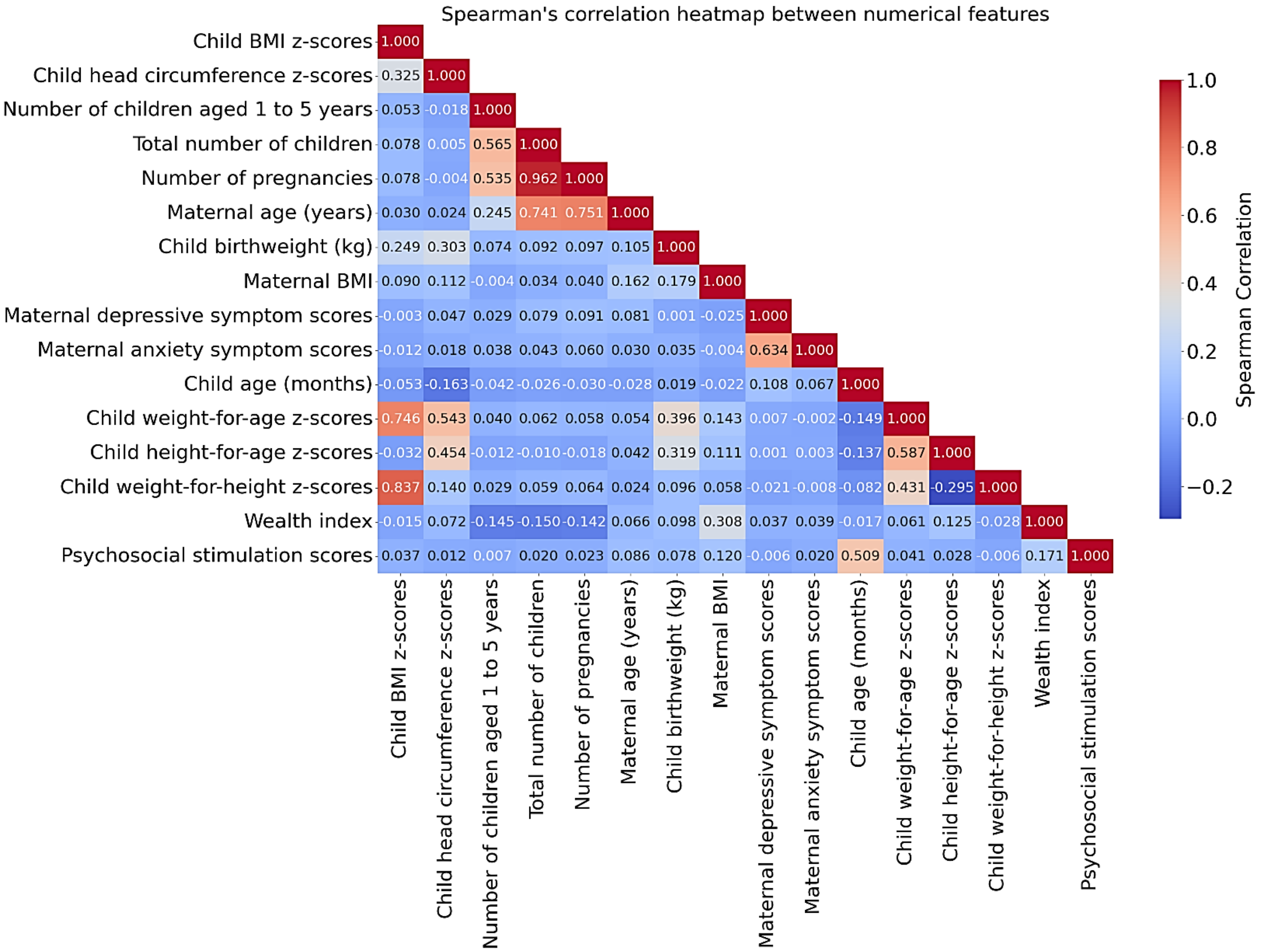

Legend: BMI, Body Mass Index.

3. Supplementary Figure S2: Correlation of categorical features

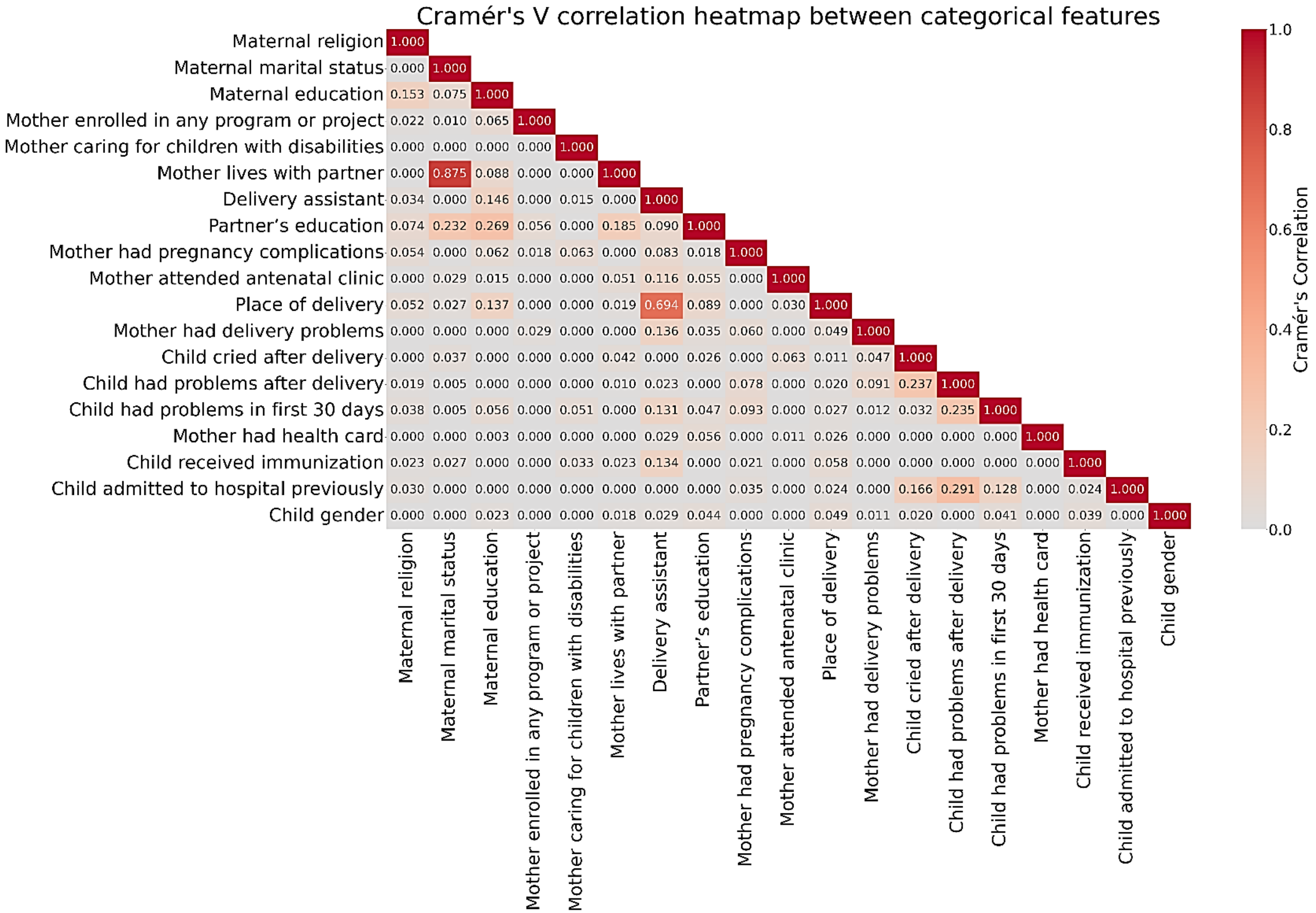

4. Supplementary Figure S3: SHAP partial dependence plots for the 15th to 20th most important predictors, by Random Forest (RF)

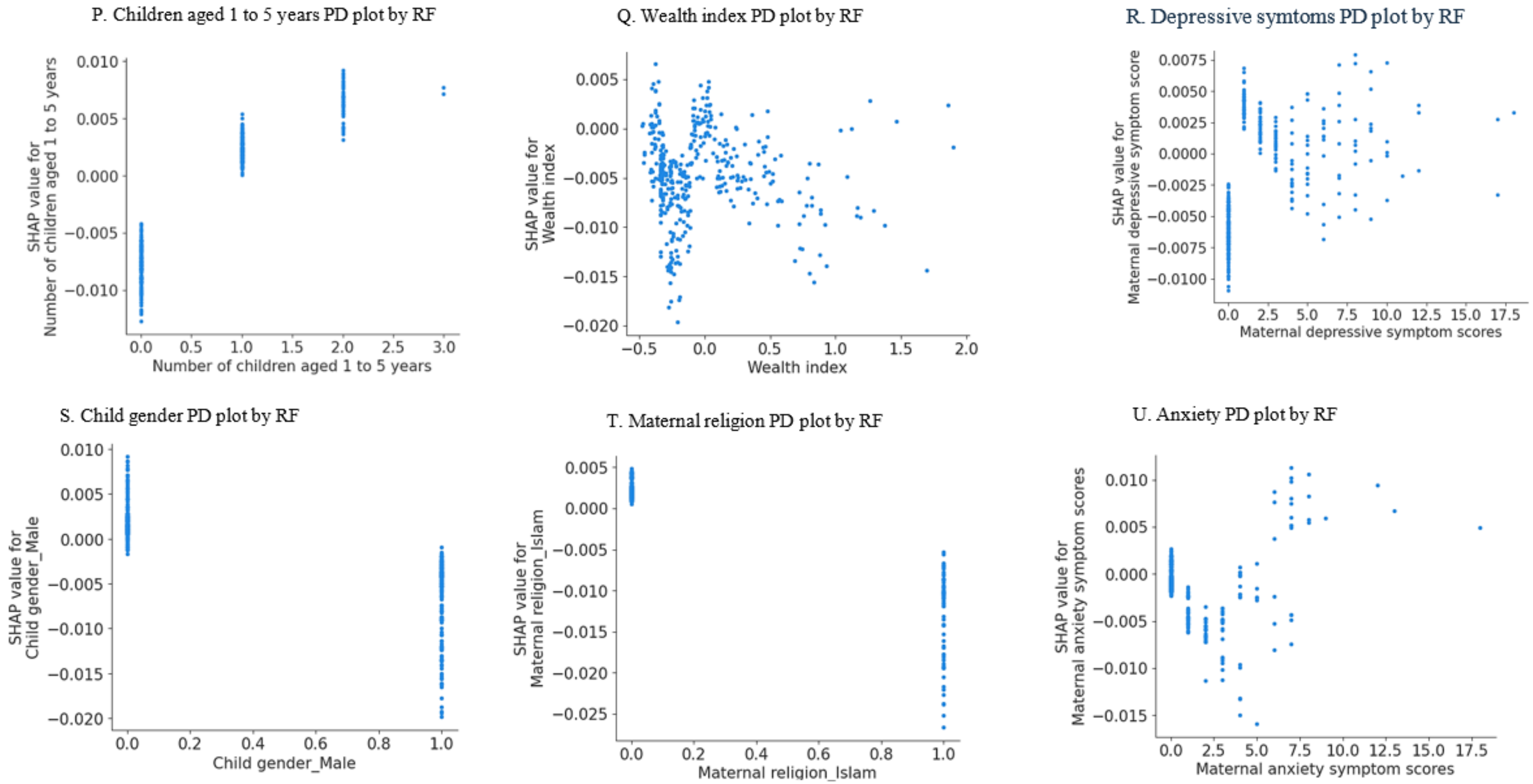

Legend: Plots P–U display the SHAP partial dependence for the 15th to 20th most important predictors. A higher number of children aged 1 to 5 per mother, low wealth index, increased depressive symptoms, being female child, not identifying as Muslim, and increased anxiety symptoms are all positively associated with off-track development.

5. Supplementary Figure S4: Interaction between place of delivery (clinic) and delivery assistant (nurse)

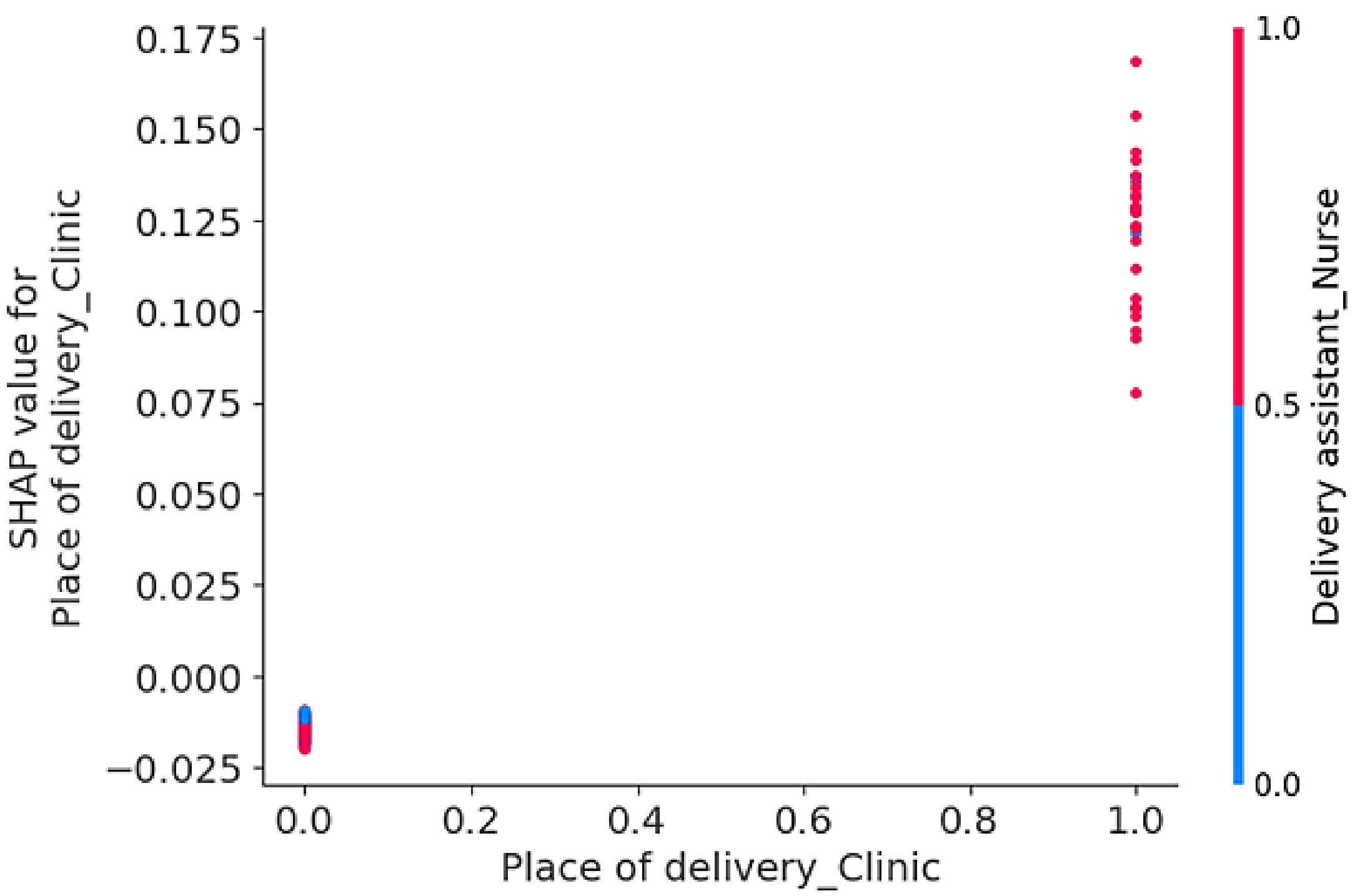

Legend: Partial dependence plot illustrating a positive interaction effect between delivery in a clinic and assistance by a nurse on the prediction of off-track development.

6. Supplementary Table S2: Features retained for the modelling process after one hot encoding

1	Maternal religion_Christian	
2	Maternal religion_Islam	
3	Maternal religion_Traditional	
4	Maternal education_No education	
5	Maternal education_Primary	
6	Partner's education_No education	
7	Partner's education_Primary	
8	Mother enrolled in any program or project_No	
9	Mother caring for children with disabilities_No	
10	Mother lives with partner_Yes	
11	Maternal marital status_Unmarried	
12	Place of delivery_Home	
13	Delivery assistant_Nurse	
14	Delivery assistant_Relative	
15	Delivery assistant_TBA	
16	Delivery assistant_Unassisted	
17	Place of delivery_Clinic	
18	Mother had pregnancy complications_Yes	
19	Mother attended antenatal clinic_Yes	
20	Mother had delivery problems_Yes	
21	Child cried after delivery_Yes	
22	Child had problems after delivery_Yes	
23	Child had problems in first 30 days_Yes	
24	Mother had health card_Yes	
25	Child received immunization_Yes	
26	Child admitted to hospital previously_Yes	
27	Child gender_Male	
28	Child age (months)	
29	Child weight-for-age z-scores	
30	Child height-for-age z-scores	

31	Child weight-for-height z-scores	
32	Wealth index	
33	Maternal age (years)	
34	Child birthweight (kg)	
35	Maternal BMI	
36	Maternal depressive symptom scores	
37	Maternal anxiety symptom scores	
38	Number of children aged 1 to 5 years	
39	Total number of children	
40	Number of pregnancies	
41	Psychosocial stimulation scores	
42	Child BMI z-scores	
43	Child head circumference z-scores	

Legend: TBA, Traditional Birth Attendant; BMI, Body Mass Index

7. Supplementary Text S1: Study tools

7.1. World Health Organisation Indicators of Infant and Young Child Development (WHO-IYCD)

The WHO-IYCD,[1] a parent-caregiver based questionnaire, was used to assess overall child development, including fine and gross motor skills, language and cognitive abilities, socio-emotional status, and general behaviour [1]. Responses for motor, language, and cognitive skills are recorded as 'Yes' (1), 'No' (0), or 'Don't know' (counted as 0). Socio-emotional and behaviour items are scored as 'Always/almost always' (2), 'Sometimes' (1), 'Never/almost never' (0), with 'Don't know' also counted as 0. Higher scores on the assessment indicate better developmental progress. Validated in Kenya, Brazil, Malawi, and Pakistan, the tool is suitable for diverse income settings, enhancing its applicability across different regions [1].

7.2. Patient Health Questionnaire (PHQ-9)

The PHQ-9[2] questionnaire was used to evaluate symptoms of depression among mothers. This tool comprises nine questions, each scored on a scale from 0 (not at all) to 3 (nearly every day). By adding up the scores from each item, a total score between 0 and 27 is obtained, where higher scores reflect more severe depressive symptoms. The PHQ-9 has been validated for use in screening depression in several studies conducted across Sub-Saharan Africa[2-4].

7.3. Generalised Anxiety Disorder (GAD-7)

Maternal anxiety symptoms were assessed using the GAD-7,[5,6] a seven-item questionnaire that captures experiences such as nervousness, excessive worry, and restlessness over the preceding two weeks. Each item is rated on a scale from 0 (not at all) to 3 (nearly every day), and the total score, ranging from 0 to 21, is obtained by summing the responses. Higher scores reflect more severe anxiety symptoms. The GAD-7 has been validated as an effective tool for screening generalized anxiety disorder[5,6].

7.4. Home Screening Questionnaire (HSQ)

The HSQ[7,8], is a parent-report tool used to assess psychosocial stimulating activities for children from birth to 6 years. It comprises 30 items and a toy checklist. The HSQ has been validated as a screening tool in low-resource settings [9]. For our study, we adapted the HSQ to better suit our context, with guidance from a domain expert. We used the 30 items in a binary format (1 and 0). Lower total scores indicate limited psychosocial stimulation in the household (e.g., few toys, limited written materials, and limited family involvement), while higher scores reflect environments rich in stimulating activities. The internal consistency of the HSQ was acceptable, with a Cronbach's alpha of 0.84 and McDonald's omega of 0·87.

7.5. Social demographic and economic questionnaire

Socioeconomic status was measured using a set of ten household assets commonly used in Kenya, including items like a radio, television, refrigerator, cooker, computer/tablet, bicycle, motorcycle, car/truck, and telephone[10]. Ownership of each asset was scored as 1, while non-ownership was scored as 0. Housing quality was also assessed based on key features such as floor, wall, and roof materials (e.g., tiles or mud for floors, bricks or mud for walls, iron sheets or grass for roofs), type of toilet facilities (e.g., flush or bush), and the location of the cooking area

(e.g., indoor or outdoor)[11,12]. A composite wealth index was then generated using Multiple Correspondence Analysis (MCA), which is appropriate for analysing binary variables[13].

7.6. Clinical and health history questionnaire

A parent-reported yes/no questionnaire collected health history data for mothers and children. For mothers, it covered pregnancy complications, delivery problems, and other health factors. For children, it included birth-related details such as crying at birth and early health issues. Birthweight and other clinical data were obtained from maternal health cards.

7.7. Anthropometric measurements

Following WHO-recommended procedures, height and weight measurements were taken from both mothers and children. To ensure quality, each measurement was taken three times. Maternal Body Mass Index (BMI) was then calculated and used as an indicator of nutritional status. Child growth indicators, including head circumference, BMI z-scores, weight-for-height z-scores, height-for-age z-scores, and weight-for-age z-scores, were calculated using the 'zscorer' package in R[14], based on WHO Child Growth Standards[15].

8. Reference

1. Gladstone M, Lancaster G, McCray G, et al. Validation of the infant and young child development (IYCD) indicators in three countries: Brazil, Malawi and Pakistan. *International journal of environmental research and public health* 2021; **18**(11): 6117.
2. Kroenke K, Spitzer RL, Williams JB. The PHQ-9: validity of a brief depression severity measure. *Journal of general internal medicine* 2001; **16**(9): 606-13.
3. Akena D, Joska J, Obuku EA, Stein DJ. Sensitivity and specificity of clinician administered screening instruments in detecting depression among HIV-positive individuals in Uganda. *AIDS care* 2013; **25**(10): 1245-52.
4. Cholera R, Gaynes B, Pence B, et al. Validity of the patient health questionnaire-9 to screen for depression in a high-HIV burden primary healthcare clinic in Johannesburg, South Africa. *Journal of affective disorders* 2014; **167**: 160-6.
5. Spitzer RL, Kroenke K, Williams JB, Löwe B. A brief measure for assessing generalized anxiety disorder: the GAD-7. *Archives of internal medicine* 2006; **166**(10): 1092-7.
6. Nyongesa MK, Mwangi P, Koot HM, Cuijpers P, Newton CR, Abubakar A. The reliability, validity and factorial structure of the Swahili version of the 7-item generalized anxiety disorder scale (GAD-7) among adults living with HIV from Kilifi, Kenya. *Annals of general psychiatry* 2020; **19**: 1-10.
7. Pessanha M, Bairrão J. The home screening questionnaire: A validation study. *International Journal of Child & Family Welfare* 2003; **6**(1-2): 27-32.
8. Frankenburg WK, Coons CE. Home Screening Questionnaire: its validity in assessing home environment. *The Journal of pediatrics* 1986; **108**(4): 624-6.
9. Nair M, Prasanna G, Jeyaseelan L, George B, Resmi V, Sunitha R. Validation of Home Screening Questionnaire (HSQ) against home observation for the measurement of environment (HOME). *Indian Pediatrics* 2009; **46**: S55-S8.
10. Abubakar A, Van de Vijver F, Van Baar A, et al. Socioeconomic status, anthropometric status, and psychomotor development of Kenyan children from resource-limited settings: a path-analytic study. *Early human development* 2008; **84**(9): 613-21.
11. Habitat U. Module 1: adequate housing and slum upgrading. *SDG indicator* 2018; **11**(1).
12. Florey L, Taylor C. Using household survey data to explore the effects of improved housing conditions on malaria infection in children in sub-Saharan Africa. 2016.
13. Booysen F, Van Der Berg S, Burger R, Von Maltitz M, Du Rand G. Using an asset index to assess trends in poverty in seven Sub-Saharan African countries. *World Development* 2008; **36**(6): 1113-30.
14. Myatt M, Guevarra E, Guevarra ME. Package 'zscorer'. *A vaila ble at: https://cran rproject org/web/packages/zscorer/zscorer pdf Accessed 3 Oct 2020* 2019.

15. Status WP. The use and interpretation of anthropometry. *WHO technical report series* 1995; **854**(9).
